# Landing mosquitoes bounce when engaging a substrate

**DOI:** 10.1038/s41598-020-72462-0

**Published:** 2020-09-25

**Authors:** Nicholas M. Smith, Jasmine B. Balsalobre, Mona Doshi, Bradley J. Willenberg, Andrew K. Dickerson

**Affiliations:** 1 Department of Mechanical and Aerospace Engineering, University of Central Florida, Orlando, USA; 2 Department of Internal Medicine, College of Medicine, University of Central Florida, Orlando, USA

**Keywords:** Biological physics, Animal behaviour

## Abstract

In this experimental study we film the landings of *Aedes aegypti* mosquitoes to characterize landing behaviors and kinetics, limitations, and the passive physiological mechanics they employ to land on a vertical surface. A typical landing involves 1–2 bounces, reducing inbound momentum by more than half before the mosquito firmly attaches to a surface. Mosquitoes initially approach landing surfaces at 0.1–0.6 m/s, decelerating to zero velocity in approximately 5 ms at accelerations as high as 5.5 gravities. Unlike Dipteran relatives, mosquitoes do not visibly prepare for landing with leg adjustments or body pitching. Instead mosquitoes rely on damping by deforming two forelimbs and buckling of the proboscis, which also serves to distribute the impact force, lessening the potential of detection by a mammalian host. The rebound response of a landing mosquito is well-characterized by a passive mass-spring-damper model which permits the calculation of force across impact velocity. The landing force of the average mosquito in our study is approximately 40 $\upmu$N corresponding to an impact velocity of 0.24 m/s. The substrate contact velocity which produces a force perceptible to humans, 0.42 m/s, is above 85% of experimentally observed landing speeds.

## Introduction

Insect flight is an enduring topic, with numerous studies on takeoff^1–3^, in-flight mechanics^4–6^, and landing^7–9^. Landings are unique from other flight maneuvers because they require matching the relative motion of a target, demanding highly-coordinated movements in response to visual, thermal, acoustic, and olfactory signals^10–15^. Landings are initiated to intercept prey, forage from dynamic surfaces, and perch to rest and nest^16,17^. Frequent feeding requires flying insects to engage dynamic targets, from flowers swaying in a breeze to mammals in motion, and the mosquito provides an example of an animal which engages both animate and inanimate nutrition sources. Mosquitoes are notorious for covertly feasting on blood, a process which begins with landing and is accomplished across a range of relative velocities and surface orientations^18^. *Aedes (Ae.) aegypti* mosquitoes are among the most prolific and dangerous mosquito species globally^19–21^, and like all mosquitoes, rely on blood meals for maturation of eggs^22,23^. Remaining undetected by the host during landing, feeding, and takeoff maximizes the probability of a successful meal. Despite their relevance to society, passive and active mechanisms by which mosquitoes initialize this process are understudied. The study of aerial landings across a variety of physiology, and under a plethora of environmental pressures, is not only imperative to understanding biological mechanisms, but may also be extended to the adaptation and survivability of emerging small unmanned aerial systems (SUAS) on the scale of insects^24^.

Mosquitoes, like many insects, navigate their environments through a combination of visual and olfactory cues and the act of host-seeking has received much attention in regard to attraction to scents^25^, patterns^26,27^, colors^28^, and illumination^29^. Historically, the vast majority of insect vision studies have been performed with fruit flies (*Drosophila melanogaster*)^30,31^. Mosquitoes possess a similar neurological construct to that of fruit flies^32^, who can be conditioned to discern different patterns with the aid of a sucrose stimulus^33^. Previous studies of mosquito vision have focused on site selection preference driven by site color (wavelength) and patterning. Gravid *Ae. albopictus* adults prefer solid black ovitraps as opposed to lighter colored ovitraps^28^. *Ae. aegypti* mosquitoes show greater responsiveness to color than the shape or pattern of ovitraps^26^. Indeed, *Ae. aegypti* prefer to land on darker surfaces of hosts and surfaces of low reflectance, supporting the assertion of their craftiness^23,34,35^. *Anopheles gambiae* females, by comparison, are capable of associative pattern recognition and able to localize a food source on both checkered and concentric patterns of black and white, according to their conditioning^27^. Landing is the final stage in host-seeking behavior, which must be completed such that the host cannot thwart the mosquito mission for a blood meal.

The landing strategies of insects differ from those of vertebrates in both timescale, distance, and speed due to highly contrasting anatomy and function. Insects’ immobile eyes and fixed focus optics prevent binocular stereopsis to gauge the distance from a substrate outright^9,36,37^. Insects instead use image motion to determine substrate distance. They monitor object expansion relative to their own motion, and control flight based on the rate of change of perceived object size^11,17^. Honeybees (*Apis mellifera*) decelerate to a hover 16 mm from a landing surface, demonstrating that touchdown is modulated through relative distance^17,38^, and initiate touchdown with legs on vertical walls whilst pitching their abdomen to dissipate residual flight energy^39^. Similarly, hawkmoths (*Macroglossum stellatarum*) decelerate upon approaching a flower and hover before initiating touchdown^40^. A female housefly (*Musca domestica*) approaches a landing surface at a constant velocity until the object reaches a critical size on its retina to induce deceleration^11^. Upon approach to a vertical landing surface, legs extend and bodies pitch upward^7^, most likely as a means to decelerate–flight velocity and pitch are inversely related in houseflies^41^. In contrast, fruit flies (*Drosophila melanogaster*) accelerate towards their landing surface and, upon touchdown, use leg forces to undergo nearly instant deceleration^8^. Legs extend prior to inverted surface landings and upon touchdown legs also assist in body rotation for multiple appendage engagement^42^.

**Figure 1:**
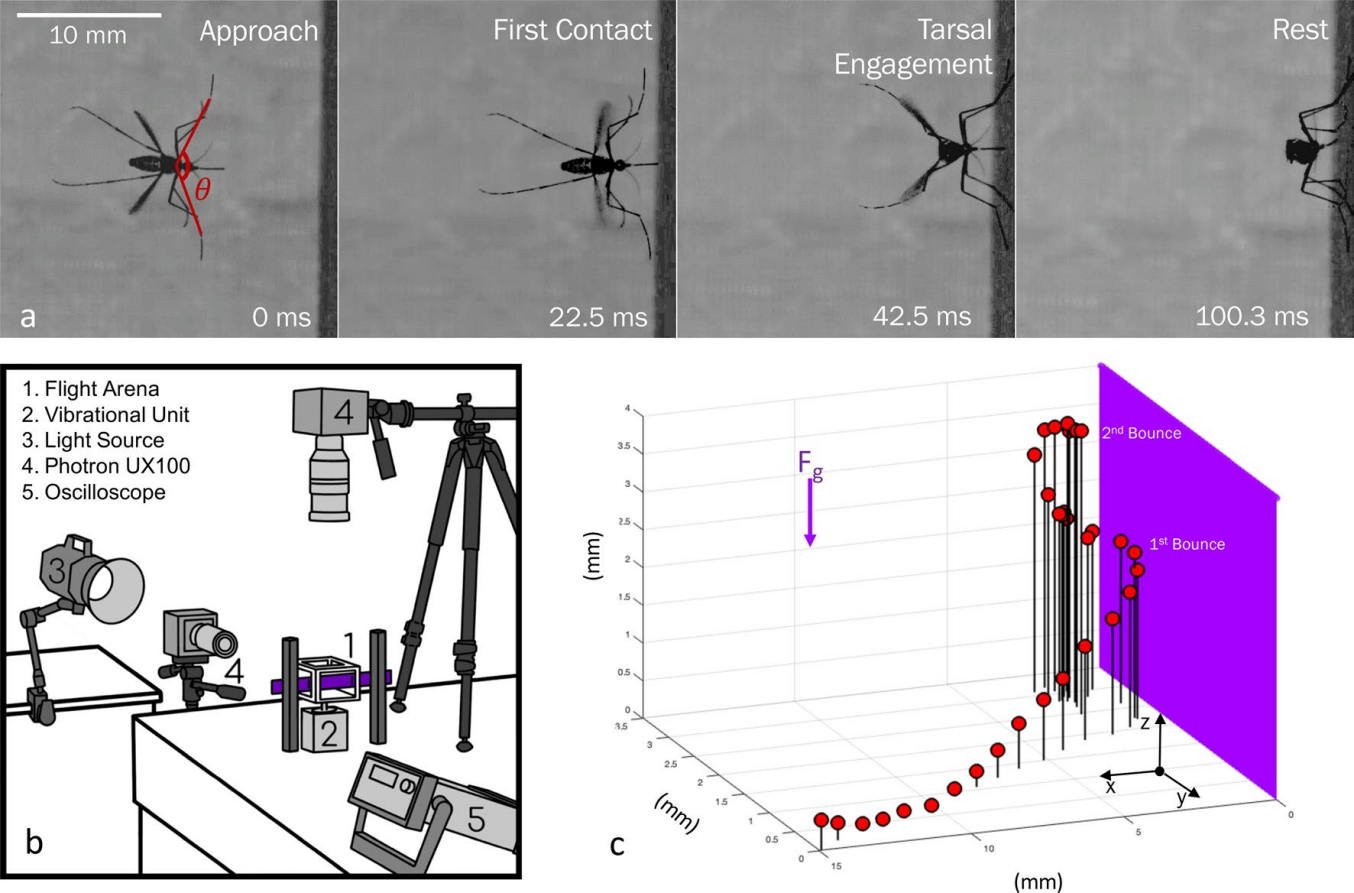
(**a**) Photographic landing sequence viewed from above. (**b**) Experimental setup of flight arena and orthogonally-positioned high-speed video cameras. (**c**) Three-dimensional displacement plot of a mosquito landing with 2.5 ms between each position marker.

Detailed *Ae. aegypti* landing mechanics are absent from literature, to the authors’ knowledge, but are likely unique from other insects due to diet, wing mechanics^4^, physiological proportions, and mass. A mosquito has 1–10% the mass of a housefly, honeybee, and hawkmoth^17,43^, and twice that of a fruit flies with dramatically different flight mechanics^44,45^. Mosquito mass allows for survival of collisions with objects of much larger mass traveling at greater speeds^46^, but the influence of mass on landing has not been studied. Typical mosquito flight posture is characterized by fore, mid, and hind-legs raised and splayed, perhaps for the sake of reducing in-flight drag^22^.

In this experimental study we reveal the mechanisms mosquitoes employ to engage hosts with landing forces below which humans can sense^47^. We observe mosquito landings with high-speed cameras, seen in Fig. 1, and digitize their motion to quantify landing forces, the employment of various appendages, and the ability of mosquitoes to cleave to surfaces across a range of contact velocity with a static surface. We begin with a description of our experimental methods in “Experimental methods” section. We initiate “Results” section with a description of the landing sequence and follow with a presentation of kinematics, forces, and energy. We discuss implications of our results in “Discussion” section, and provide concluding remarks in “Conclusion” section.

## Experimental methods

### Mosquito rearing and care

*Aedes aegypti *eggs were obtained from the United States Department of Agriculture-Agricultural Research Service, Center for Medical, Agricultural and Veterinary Entomology (USDA-ARS-CMAVE, Gainesville, FL) and continued to be cultivated in the Willenberg Lab as described elsewhere^48^. Briefly, 8 mg of eggs ($\sim 800$ eggs) are brushed off the cards and shaken vigorously in a glass vial containing 7.5 mL of larval food in deionized (DI) water (40:60; brewer’s yeast and liver powder). The solution is transferred into 3 L of DI water in a plastic tray. These trays are incubated at 29–30 $^\circ$C and larval food is added at day 3 (7.5 mL), day 4 and day 5 (10 mL). At day 6, the larvae/pupae are poured over a 500 $\upmu$m strainer, rinsed with fresh DI water and transferred to 200 mL of DI water in 237-mL (50 cm$^2$ surface area) cups. These cups are placed in a ($20.3\times 20.3$)-cm rearing cage and kept in a 29–30 $^\circ$C incubator. Within 24 h, most mosquitoes emerge and this counts as day post-emergence (DPE) one. Sucrose (10% w/v) is provided *ad libitum* via a saturated cotton ball placed atop the rearing cages. On DPE 3, the mosquitoes are cold anesthetized in a cold room for 30 min. The mosquitoes were then collected in a cup and kept on ice. As required, 50 male and 50 female mosquitoes are sorted into separate cups by spreading them on a plate kept on ice and grasping the hind legs for transfer. The cups are covered with screen mesh and 10% (w/v) sucrose provided *ad libitum* through small saturated cotton balls.

### Landing experiments

We film 20 landings of non-blood-fed female *Ae. aegypti* mosquitoes onto a rigid, vertical surface. Landings are filmed within a plastic 3D-printed flight arena lined with acrylic walls. The arena internally measures ($70 \times 100 \times 140$) mm (H $\times$ W $\times$ L), as seen in Fig. 1b. A purple substrate, the width of the container and 40 mm in height, is placed at one end of the arena to serve as the landing surface; darker hues elicit landings at a rate 9x higher than clear or white substrates^29^. Mosquitoes are anesthetized with CO$_2$ for placement into the flight arena, and given sufficient time to recover from anesthetization before filming. To encourage resting mosquitoes into flight, the arena is vibrated at low amplitude 25 Hz for up to 5 s. Vibration does not catapult mosquitoes from walls and mosquitoes are given at least 1 s ($\sim 600$ wingbeats)^2^ to recover flight before a landing is considered for analysis. The landing surface protrudes through the walls of the arena and is supported externally such that it does not vibrate with the arena walls. After cessation of vibration, only landings that originate from an orthogonal distance greater than 10 mm is saved for analysis to exclude landings influenced by adjacent wall or neighbor mosquito contact in the moments preceding landing. Landings were recorded on six individual days. Fifty mosquitoes are replaced into the container simultaneously after which multiple landings are recorded—3, 4, 7, 1, 2, 3 landings per recording day, respectively. A new group of 50 mosquitoes is used each filming day. We treat all landings as independent samples and acknowledge the probability of one pseudo-replicate for the entire experimental dataset is 24.6%.

### Filming

Landings are filmed using Photron AX-100 and UX-100 high-speed cameras at (2000–4000) fps in single and dual camera configurations. All reported kinematic measurements are extracted from single camera experiments for the sake of measurement precision. We view landings from above with a 150-mm Nikon lens and measure the position and velocity in a horizontal plane orthogonal to the landing substrate. Mosquito kinematics are digitized with Open Source Physics Tracker (OSPT) by tracking where the proboscis meets the head in each video frame. OSPT is calibrated with a grid of known dimension in frame and tracks the spatial position of a specified point on the mosquito across frames. Two cameras are utilized for 3D reconstruction to visualize incoming flight path and bouncing sequences as shown in Fig. 1c. In two camera experiments, an additional camera is placed to view the landing surface in its entirety. The cameras are fitted with 60 mm (side) and 24–120 mm (top) Nikon lenses. 3D trajectories are extracted from paired videos with direct linear transformation, DLTdv6^49^. Wing rotation values are taken from videos not used in extracting landing kinematics but instead view mosquitoes top-down.

### Physical characterization

The proboscis is modeled as an end-loaded cantilever beam. Three proboscises were excised from the head and affixed to a rigid rod with UV-curable glue and filmed within 10-min of excision. A Keyence VHX-900 digital microscope, which has internal pixel calibration, is used to measure proboscis diameter and cantilevered length at a magnification of 150x. The Keyence VHX-900 also films the accumulation of water produced by an ultrasonic humidifier. All deposited moisture except a single droplet is removed manually before measurement. The deflection of the proboscis under the weight of the single droplet is measured optically by photographic measurements in OSPT. The experiment was replicated twice for each proboscis to achieve an average value. Damping characteristics are determined by using a modified cubic flight arena of characteristic length $37.5$ mm to inhibit free flight and ground the mosquitoes. The mosquitoes are vibrated at a fixed frequency of 25 Hz and 50 Hz for a few seconds to establish sinusoidal behavior, and then vibration is ceased. Videos are analyzed with OSPT.

## Results

### Description of landing and orientation preference

**Figure 2:**
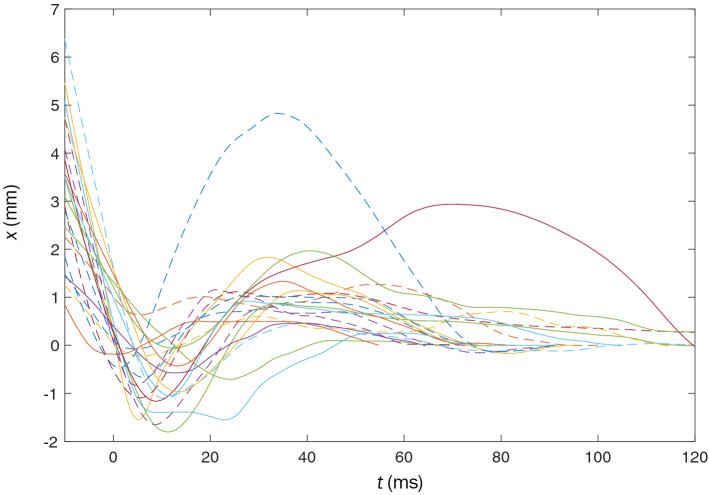
Normal-to-substrate displacement for all 20 analyzed landings. The tracked point on the mosquito is the interface of the proboscis with the head. The final resting position of the tracked point corresponds to $x=0$. First contact of any portion of the mosquito with the landing surface corresponds to $t=0$. Dashed-curves indicate the proboscis is the first member to contact the substrate, while solid lines indicate tarsi initiate contact. Curves are smoothed with a second-order Savitzky–Golay filter at 10% span.

**Figure 3:**
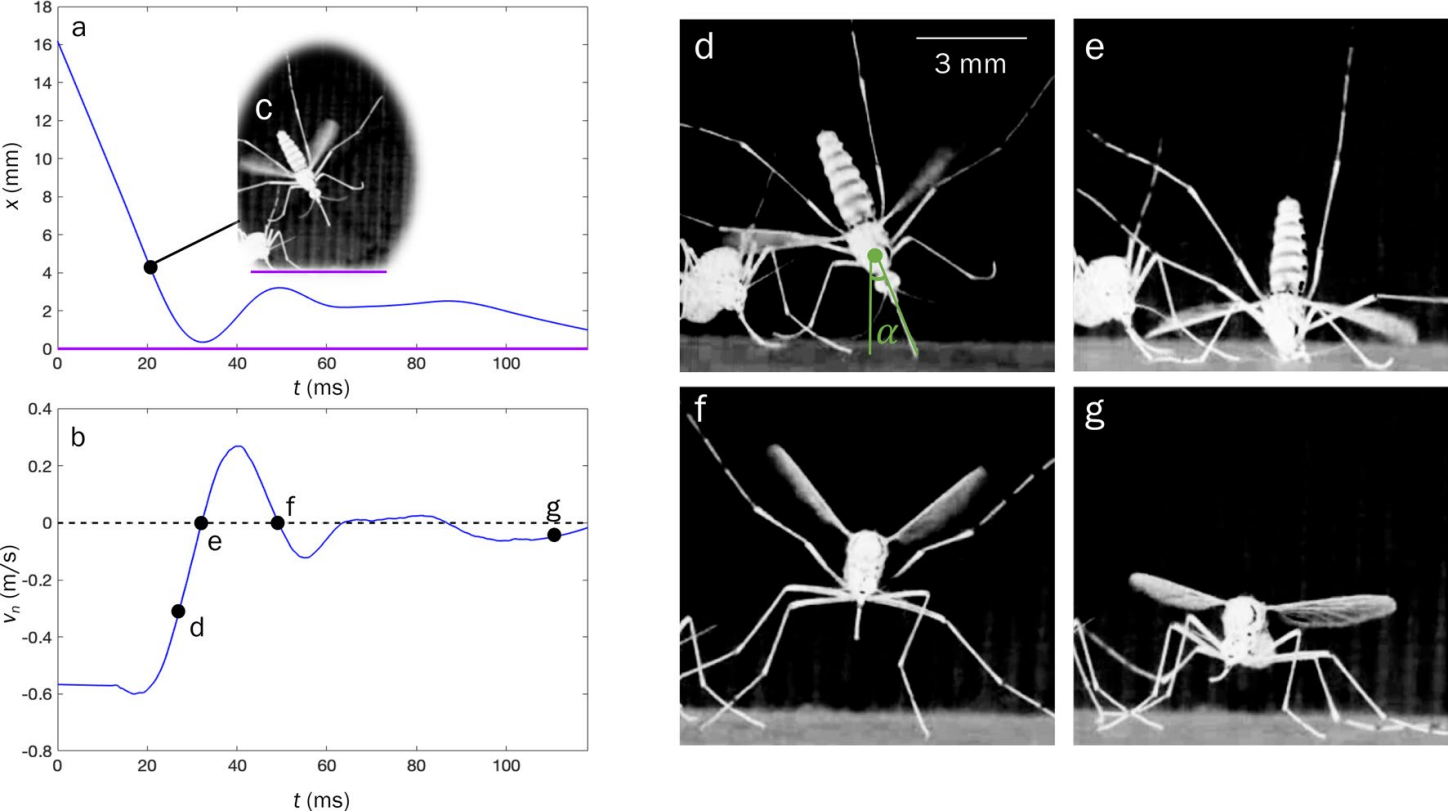
Mosquito landing plots for (**a**) Temporal normal-to-substrate displacement. (**b**) Temporal normal-to-substrate velocity. Data in plots (**a**) and (**b**) is smoothed with a second-order Savitzky–Golay filter at 10% span. (**c**) Initial deceleration of mosquito. (**d**) First contact of proboscis. (**e**) Collapse of the proboscis with head nearly contacting surface. (**f**) Maximum bounce displacement. (**g**) Final stabilization of landing position with eminent wing retraction.

The walls of the flight arena are briefly vibrated to encourage flight of resting mosquitoes. Mosquito landings are considered for analysis if a forward appendage (forelimbs or proboscis) initiates contact with the landing surface. All analyzed landings are shown in Fig. 2 as smoothed curves and in Fig. S1 as raw curves. Temporal velocity for all landings are is plotted in Fig. S2. Mosquito flight posture is characterized by both forelimbs projecting outward with $\theta _\text {legs}=118.4 \pm 8.1^\circ$, $\text {N}=5$ with respect to one another if measured from the thorax dorsal center. Typical flight posture is shown in Fig. 3a,c (Movie S1). Such a foreleg posture avoids lateral engagement of substrates up to an angle of incidence $\alpha = \theta _\text {legs}/2 = 59.2 \pm 4.1^\circ$. We limit our scope of analysis to landings in which the angle of incidence of approach is less than $\alpha$ to eliminate landings which were slowed, or otherwise influenced, by grazing contact of aft legs and wings prior to substrate engagement.

Mosquitoes approach the test surface with a normal velocity $v_\text {n} = -\,0.24 \pm 0.14$ m/s, $\text {N}=20$, as shown in Fig. 2 for $t<0$. Upon tarsal contact with the substrate specimens rapidly decelerate, shown graphically in Figs. 2 and 3a, b. Sensing of the substrate prior to touchdown is likely done with a combination of vision and self-induced pressure wave detection^50^. Encounters with the substrate intermittently occur proboscis first, shown in Fig. 3d, and produce compression of the forelegs and often buckling/deformation of the proboscis, lengthening impact time, seen in Fig. 3e. Mosquitoes bounce from the surface at a normal velocity $0.16 \pm 0.08$ m/s, N $=$ 19, and reverse course back toward the substrate at an average distance of 1.7 mm. A bounce is witnessed when the torso experiences movement away from the substrate. Landings in which tarsi do not separate from the substrate following initial contact display a single bounce. A double bounce landing is plotted in three dimensions in Fig. 1c, two dimensions in Fig. 3a, and pictured in Fig. 3d–g. Only a single trial displayed no bounce. Mosquitoes display a bounce pattern which ceases when tarsal grip is sufficient to overcome bounce acceleration, within 3 bounces (Movie S2). Every bounce and subsequent approach acts to reduce the mosquitoes’ incoming momentum by at least 50%, N $=$ 19. With forelimb tarsi securely in place, the abdomen and remaining legs swing downward to contact the surface as the wings cease flapping. Once coming to their resting position, Fig. 3g, wings then rotate inward at an average angular velocity $12,977 \pm 4844\,{^{\circ }}$/s, $\text {N}=5$ (left wing) and $12,943 \pm 4932\,{^{\circ }}$/s, $\text {N}=4$ (right wing) to rest atop the abdomen approximately 100 ms after approach, as pictured in Fig. 1a.

While we analyze only landings onto vertical surfaces in this study, we do quantify the frequency of landings onto vertical and horizontal surfaces within our flight chamber. Over the time-course of 5 minutes, beginning at the cessation of arena vibration, we count the number of landings onto the purple substrate when oriented vertical and horizontal, in separate trials. For each trial, 50 female mosquitoes were placed in the arena simultaneously. We count $29 \pm 3$, $\text {N}=3$, landings on the vertical surface and a meager $3 \pm 2$, $\text {N}=3$, landings on the horizontally oriented surface, a result which is in line with previous observation of mosquito preference^19^. We note that for many mosquito hosts, humans for example, vertically oriented surface area exceeds that of horizontally-oriented surface area.

### Impact energy and proboscis bending

**Figure 4:**
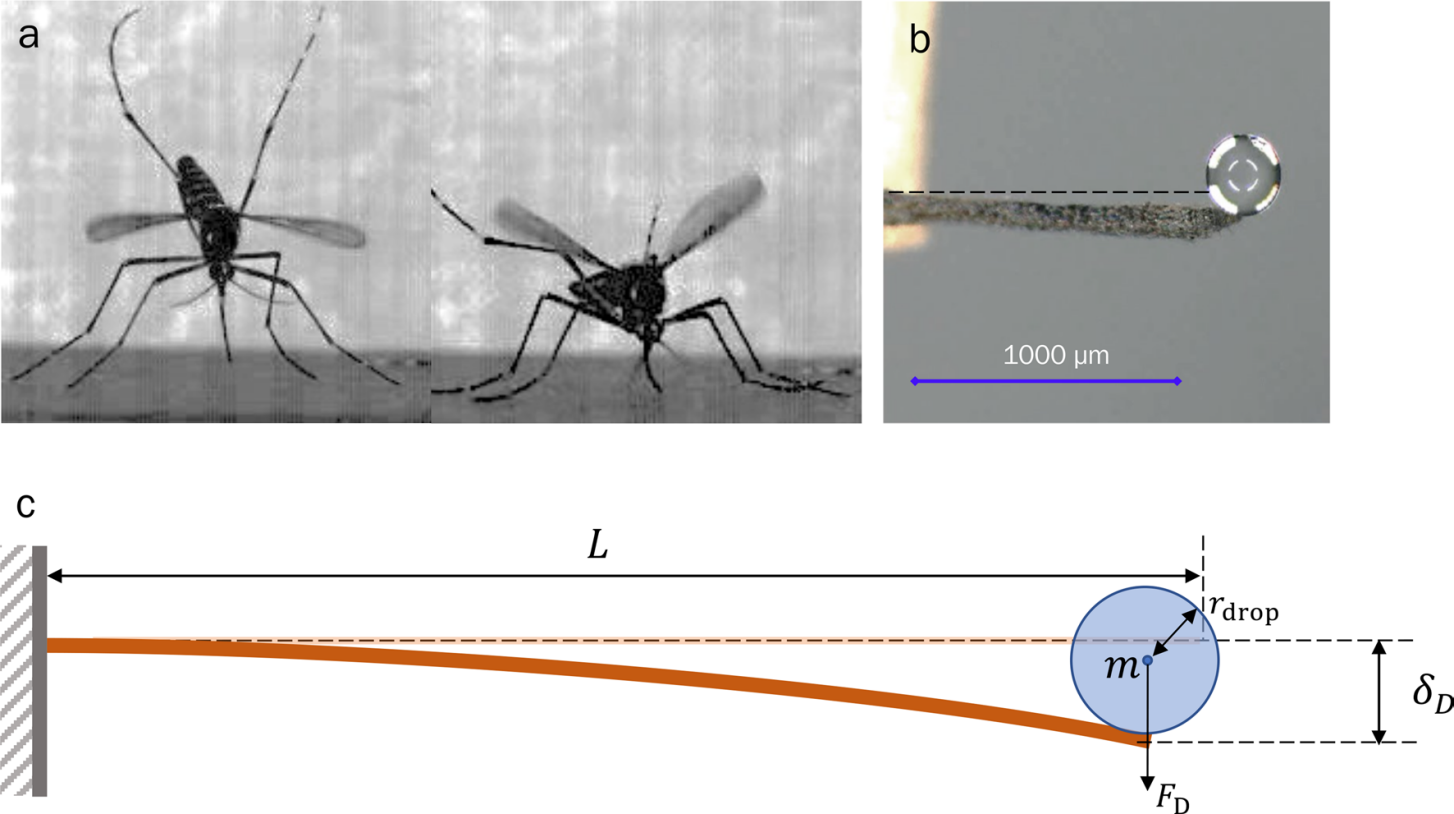
Mosquito landing with proboscis (**a**) initiating contact with substrate, and deflecting from normal force. (**b**) Modulus experiment with mosquito proboscis fixed on one end and loaded on free end with a water droplet. (**c**) Diagram of measured parameters depicting proboscis deflection due to end load.

As legs compress, wings flap, and proboscises deform, mosquitoes absorb their in-flight kinetic energy $E_\text {k} = m v_\text {n}^{2}/2=0.061~\upmu$J, where the average mosquito mass $m=1.66$ mg, N $=$ 30. In the absence of detailed wing kinematics and computational fluid dynamics, parsing the energy absorbed in the legs $U_\text {l}$ from that absorbed by the wings $U_\text {w}$ is not feasible, and is thus beyond the scope of the current study. Therefore, we quantify the energy absorbed $U_\text {p}$ via proboscis deflection $\delta _\text {p}$, preceding the initial bounce, seen in Fig. 4a, b (Movie S3). Proboscis deflection is not seen in subsequent bounces and was present in 16 of 20 recorded landings. Altogether we may write $E_\text {k} = U_\text {p} + U_\text {l} + U_\text {w}$, and note that potential energy is neglected in our consideration of conservation of momentum in the direction perpendicular to the landing surface.

Deflection of the proboscis $$\delta _\text {p}$$ is measured approximately 5–80%, $$\text {N}=16$$, of proboscis length $$L=1.0-2.1$$ mm. We acknowledge this degree of deformation very likely places the proboscis outside the linear-elastic regime. However, to gain an understanding of the role proboscis deformation plays in the landing process without knowing precise deformed curvature, we employ linear-elastic assumptions. We model the proboscis as an end-loaded cantilever beam where the proboscis deflection stores elastic strain energy $$U_\text {P} = k_\text {eff}\delta _\text {p}^{2}/2$$ and force is applied normal to the beam axis. The effective stiffness of the proboscis $$k_\text {eff}$$ can be written in terms of elastic modulus $$E_\text {p}$$, area moment of inertia $$I = \pi r^4 /4 = {2.16} \times 10^{-6}\,{\hbox {mm}^4}$$, and *L*, such that $$k_\text {eff}=3E_\text {p}I/L^{3}$$, where proboscis radius $$r=43\pm 2$$$$\upmu$$m, $$\text {N}=3$$. The elastic modulus of the proboscis is determined by measuring its deflection $$\delta _\text {D}$$ under the weight $$F_\text {D}$$ of a droplet (see “Experimental methods” section), such that

1 $$\begin{aligned} E_\text {p} = \frac{F_\text {D}\ell ^3}{3I\delta _\text {D}}, \end{aligned}$$

where the cantilevered proboscis length $\ell = 917 \pm 97\,\upmu$m, $\text {N}=3$ and $\delta _\text {D}= 3.2 \pm 1.3\,\upmu$m, $\text {N}=3$. Deflection of a proboscis by a drop can be seen in Fig. 4b, and schematized in Fig. 4c. We measure $E_\text {p} = 1.56 \pm 0.16$ MPa, $\text {N}=3$, and from above,

2 $$\begin{aligned} U_\text {p} = \frac{3E_\text {p}I\delta _\text {p}^2}{2L^3}. \end{aligned}$$

For the maximum observed value of $\delta _\text {p} = 0.8L$ and $L=1$ mm, $U_\text {p}=0.0032 ~\upmu$J. In the most extreme cases the proboscis is able to absorb up to $U_\text {p}/E_\text {k} \approx 5.4\%$ of the kinetic energy of the average mosquito approach.

If instead we consider an axially loaded proboscis, the critical buckling load $P_\text {cr}$ required to produce tip movement $\delta _\text {p}$, analogous to buckling a column,

3 $$\begin{aligned} P_\text {cr} = \frac{\pi ^2 E_\text {P}I}{4L^2}, \end{aligned}$$

we calculate $P_\text {cr} = {8.3}\,{\upmu \hbox {N}}$, well below the human detection threshold, 70 $\upmu$N^47^. The exact energy calculation associated with buckling would require extensive post-buckling analysis and is complicated by complex material behaviors at large deformation. Such characteristics are not known for proboscises. Recent research in the crushing of slender structures indicates a rapid collapse of load bearing capacity at the onset of instability for even complex structures under both axial and bending loads^51,52^. Conservatively, we assume linear force *P* degradation such that $P=P_\text {cr}$ at loading onset and $P=0$ at complete collapse. The energy transferred to the proboscis is the sum of the collapse energy $U_\text {col}\approx P_\text {cr}L/2=0.0042\,\upmu$J and the assumed negligible elastic energy. Thus, $U_\text {col}/E_\text {k}\approx 6.9\%$. The agreement in values of $U_\text {p}$ and $U_\text {col}$ indicates the primary mechanisms for dissipating energy associated with orthogonal flight motion are leg compression and wing aerodynamics, discussed in “Impact force mitigation by foreleg properties” section.

### Impact force mitigation by foreleg properties

**Figure 5:**
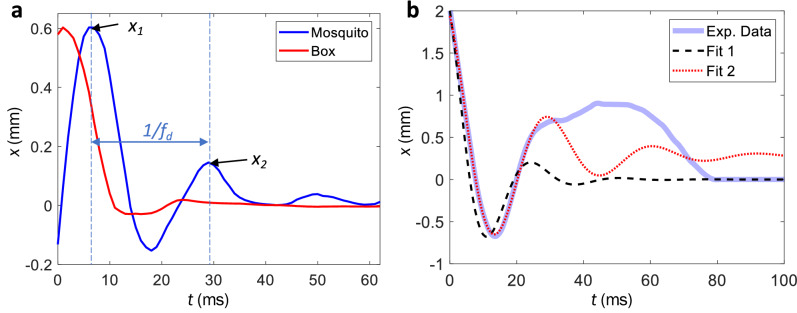
(**a**) Mosquito displacement over time for a mosquito standing on the floor of a box vibrating at 50 Hz. The curve is smoothed with a second-order Savitzky–Golay filter at 10% span. (**b**) Experimental landing data, Fit 1 based on Eq. (6), and Fit 2 from Eq. (7).

Foreleg compression at touchdown lengthens impact time and reduces impact force by distributing momentum across multiple joints. Modeling the mosquito as a simple mass-spring-damper, where the legs act as the damped spring, allows for the determination of their effective damping coefficient *c* and stiffness *k* for comparison to those ideal for force reduction. To characterize dynamic response we vibrate the box floor beneath standing mosquitoes at a fixed frequency, 25 and 50 Hz. Upon nearly impulsive cessation of floor movement we measure free response of mosquito bodies and solve the corresponding equation of motion,

4 $$\begin{aligned} {\ddot{x}}+\beta {\dot{x}}+\omega _n^2 x = 0, \end{aligned}$$

where $\beta = c/m$ and $\omega _\text {n} = \sqrt{k/m}$. The amplitude reduction factor provides the damping ratio $\zeta$ of the mosquito,

5 $$\begin{aligned} \ln {\frac{x_1}{x_2}}=\frac{2n\pi \zeta }{\sqrt{1-\zeta ^2}}, \end{aligned}$$

where *n* is the number of cycles between amplitude measurements $x_1$ and $x_2$ equal to unity in our system (Fig. 5a). Solving Eq. (5) with both 25 and 50 Hz responses, $\zeta _\text {exp} = 0.36 \pm 0.10$, $\text {N}=8$, indicating the mosquito behaves as an underdamped system, explaining mosquitoes’ propensity to bounce after first contact. The natural frequency of the mass-spring-damper analog $\omega _\text {n} = \omega _\text {d}/\sqrt{1-\zeta ^2}= 256 \pm 39$ rad/s, where $\omega _\text {d}= 239 \pm 36$ rad/s is measured from spatio-temporal data. The spring constant $k = m\omega _\text {n}^2= 0.109 \pm 0.002$ N/m, the critical damping coefficient $c_\text {c}=\sqrt{4mk}= 8.5 \pm {1.2}\times 10^{-4}$ N-s/m, and the actual damping coefficient $c = \zeta c_\text {c}= 3.1 \pm {0.5}\times 10^{-4}$ N-s/m. The general solution to Eq. (4) is,

6 $$\begin{aligned} x(t) = e^{-(\beta /2)t}[A\sin (\gamma t)+B\cos (\gamma t)], \end{aligned}$$

where $\gamma = \frac{1}{2}\sqrt{4\omega _\text {n}^2-\beta ^2}$, $A= [\beta x(0)/2+{\dot{x}}(0)]/\gamma$, and $B=x(0)$. Using the aforementioned values of *k* and *c*, we plot Eq. (6) in a dashed-black line next to a temporal track of a typical landing event, in Fig. 5b, matching the initial condition $x(0)=2$ mm of the data. We note reasonable agreement with experimental data through the first 20 ms of landing. Equation (6) does not capture the influence of aerodynamic damping of the wings, the wing-in-ground effect, and potential coulomb damping in the joints. Moreover, Eq. (6) predicts the mosquito will accelerate slightly, a consequence of modelling legs as damped, outstretched springs.

An improved fit may be garnered by prohibiting the virtual spring in the mosquito leg to be extended prior to impact, adding a constant bias *C*, and phase shift $\phi$,

7 $$\begin{aligned} x(t) = C+De^{(-\zeta \omega _\text {n}t)}\sin (\omega _\text {d}t+\phi ). \end{aligned}$$

Equation (7) is fit to the raw experimental data in Fig. 5b with a nonlinear least squares solver, where *C*, *D*, $\zeta$, $\omega _\text {n}$, $\omega _\text {d}$, and $\phi$ are free parameters. We plot the best fit provided by Eq. (7) with a red curve in Fig. 5b. The resulting $k=0.07$ N/m, $c={1.6}\times 10^{-4}$ N-s/m, and $\zeta = 0.23$ agree with those calculated from experiments of mosquito free vibration following an impulsively-stopped vibrating floor [Eq. (5)].

By setting $B=x(0)=0$ in Eq. (6) and taking the second time derivative we produce an equation for temporal substrate force that utilizes *k*, *c*, and $\zeta$ calculated through free vibration experiments,

8 $$\begin{aligned} F(t) = mAe^{-(\beta /2)t}\left[ \frac{1}{4} \beta ^{2} \sin (\gamma t)-\gamma ^{2}\sin (\gamma t)-\beta \gamma \cos (\gamma t) \right] . \end{aligned}$$

**Figure 6:**
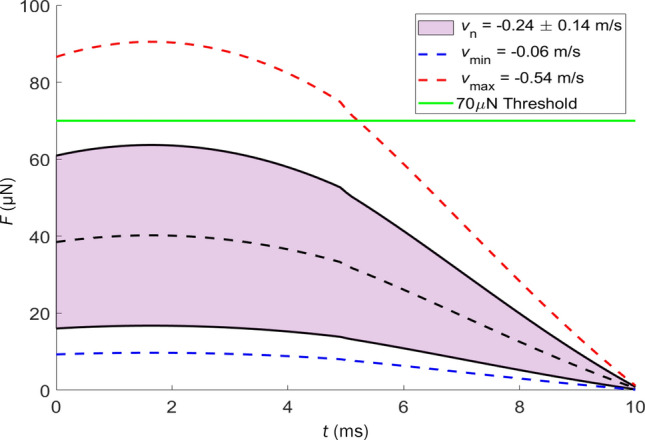
The temporal substrate force from Eq. (8) for various landing velocities. Blue and red curves represent the slowest and fastest observed velocities, respectively, while the purple cone denotes the standard deviation around the average observed velocity.

A range of ${\dot{x}}(0)=v_\text {n}$ is used to plot *F* against *t* in Fig. 6. We plot only the first 10 ms, sufficient time for rebound to begin, as seen in Fig. 5b. We assume the mosquito distributes the load uniformly between two front legs and neglect aerodynamic effects. The slowest mosquito landing provides an impact acceleration of 0.6 gravities, while the fastest impact produces 5.5 gravities. The range of landing velocities in our study is in agreement with in-flight velocities recorded in other studies^53–55^. The landing force of the average mosquito in our study is approximately 40 $\upmu$N, falling short of human detection. However, covert landings are not universal as 3 trials ($15\%$) record a magnitude of normal velocity greater than that which meets the human force detection threshold^47^, 0.42 m/s. This result aligns with authors’ experience of occasionally sensing a landing mosquito, less common than sensing the mosquito bite.

## Discussion

Our study reveals *Ae. aegypti* mosquitoes employ bouncing sequences, leg compression, and proboscis deformation to engage landing surfaces. Unlike bees^38^, houseflies^7^, and fruit flies^8^, we do not witness mosquitoes prepare for landing by adjusting leg posture or body rotation. Their substrate interactions often have head and torso contact with the substrate (Fig. 3e), but the associated forces are easily survivable and relatively small in the insect realm^46^. Proliferation mandates that landings are completed discreetly, below that which a host can sense, so that blood meals are completed unencumbered. Thus a mosquito employs multiple appendages to scrub momentum and reduce the force imparted by any one member. Any flyer, biological or engineered, aiming to land discreetly may control the effective length $x_\text {eff}$ of impact over which flight is slowed under constant acceleration. Rearranging the equation of motion and assuming no reactions other than those provided by the substrate,

9 $$\begin{aligned} x_{eff } = mv_n^2/F', \end{aligned}$$

where $F'$ is the landing force not to be exceeded. For mosquitoes we calculate $x_{eff } = 1.4 \pm 0.5$ mm for initial impact if using $F'=70\,\upmu$N. This value of $x_\text {eff}$ would be traveled in $\sim$6 ms, is $\approx 23\%$ of a mosquito body length^22^, and $\approx 33\%$ of the mosquito foreleg, appears exceedingly achievable. Yet, we observe greater compression distances by the proboscis alone, $\delta _\text {P} = 1.71$ mm, an observation that may be tied to insect perception rather than kinetics. However, it was recently discovered mosquitoes can sense sound pressure waves generated by their flapping wings rebounding from nearby surfaces, a sensory cue that is used to divert from unavoidable surfaces^50^.

While the timescale over which landings occur is rapid, it is comparable to the timescale of takeoff^2^ and lengthy compared to the timescale of a single wingbeat^56^. Thus, it is possible leg compression at landing is not wholly passive. Active engagement of leg muscles may contribute to the discrepancy between our passive model and experimental response of a landing mosquito. An active force modifier may be added to Eq. (4) to better match mosquito responses, but the magnitude and time-response of such a force is currently unknown and an area for future work. Regardless of active contribution by legs to slow the mosquito, a passive model well-describes mosquito landing.

The spread posture of mosquito legs during flight (Fig. 1a), the same as upon landing approach, may serve a purpose beyond the previously proposed drag reduction benefits^22^. The oblique angle between the two forward tarsi, $\theta _\text {legs}$, ensures that approach angles $\alpha \lesssim 60^\circ$ toward a vertical surface result in the contact of both forward tarsi. The engagement of the tarsi closest to the substrate induces body rotation to produce foreleg-substrate contact within 8.2 ms. The pliability of a proboscis to absorb impact energy is meager in comparison to the complementary work of legs and wings, and does not obstruct the foreleg tarsi from contacting the surface. The low critical force for proboscis buckling, $P_{cr}$, allows the mosquito proboscis to collapse in the tangential direction at velocities well below the average value of $v_\text {n}$.

We cannot confirm landings onto hosts are representative of those captured in this study, which may be described as controlled crashing. Mosquitoes use a variety of thermal, olfactory, and self-induced airflow cues in addition to vision to track their hosts^50,57^, but it is unclear how non-visual cues aid in clandestine landing. Mosquitoes are also nocturnal, avoiding obstacles invisible to their compound eyes^58^. We have witnessed activity in response to human attractants to be rather uncontrolled crashes when mosquitoes probe nets for passage^19^, suggesting such behavior has no landing intent. Mosquitoes are likewise more qualitatively attracted to purple than the polished and translucent acrylic trialed in preliminary experiments, and previous literature suggests they can easily distinguish solid colors from patterns^23,26,34,35^. The 40-mm high purple landing strip should stand out against its background and change in size as the mosquito approaches. If host landings differ from those on our surface, we expect they produce smaller substrate forces than we calculate as mosquitoes more adequately prepare for impact.

## Conclusion

In this study we find *Ae. aegypti* mosquitoes experience bouncing when engaging surfaces to disperse in-flight momentum. In the first bounce, a mosquito will decrease its impact velocity by approximately 50%, and passively rotate its body, by virtue of its in-flight posture, to engage both pairs of fore- and mid-legs. Landings occur in approximately 100 ms from first contact to wing retraction, and are accompanied by proboscis deflection, which crumples as mosquitoes strike surfaces at an average normal-to-substrate speed of 0.24 m/s. The proboscis is able to absorb up to $5.4\%$ of the mosquitoes’ initial kinetic energy. Thus, wing aerodynamics and leg compression are the primary mechanisms for kinetic energy dissipation. By treating the mosquito as a simple mass-spring-damper, we find a damping ratio of $0.36 \pm 0.10$, indicating mosquitoes behave as an underdamped system when engaging a surface, explaining their propensity for bouncing after their initial, and occasionally, subsequent impacts. Free vibration analysis and the assumption of uniform load distribution among both forelegs indicates landings with normal-to-substrate speeds below 0.42 m/s are undetectable by humans. The landing force of the average mosquito in our study is approximately 40 $\upmu$N corresponding to an impact velocity of 0.24 m/s.

## Supplementary information

Supplementary Information.

Supplementary Movie S1.

Supplementary Movie S2.

Supplementary Movie S3.

## Data Availability

Raw experimental videos and data are available in perpetuity via Open Science Framework: https://osf.io/9wkuj/.
