# Supplementary material for: Landing mosquitoes bounce when engaging a substrate
Source: Sci Rep. 2020 Sep 25;10:15744. doi: 10.1038/s41598-020-72462-0 (PMC7519040; doi:10.1038/s41598-020-72462-0)
Supplement: Supplementary file 1 — Supplementary Information. [file 41598_2020_72462_MOESM1_ESM.pdf]

Online Supplement for *Landing mosquitoes bounce when engaging a substrate*

Nicholas M. Smith[1], Jasmine B. Balsalobre[1], Mona Mathew[2],
Bradley J. Willenberg[2], Andrew K. Dickerson[1]*
[1]Department of Mechanical and Aerospace Engineering,
[2]Department of Internal Medicine
University of Central Florida
*Corresponding author: dickerson@ucf.edu

Supplementary movie captions

Movie S1: A female *Ae. aegypti* mosquito landing on a purple substrate with negligible tangential velocity and experiencing minor proboscis deformation. Tarsi do not leave the substrate following first contact, resulting in a single bounce. Slowed 133x.

Movie S2: A female *Ae. aegypti* mosquito landing on a purple substrate with significant tangential velocity, body rotation, and large proboscis deformation. Tarsi leave the substrate after initial contact, resulting in two bounces. Slowed 133x.

Movie S3: A female *Ae. aegypti* mosquito landing on a purple substrate with large proboscis deformation and collapse. Slowed 133x.

Supplementary figures and table

Table S1: Free vibration properties of mosquitoes measured following the impulsive cession of a vibrating floor at 25 and 50 Hz.

	25 Hz (N = 4)	50 Hz (N = 4)
ζ	0.44 ± 0.03	0.28 ± 0.06
k (N/m)	0.118	0.103
c (N-s/m)	3.9×10^{-4}	2.3×10^{-4}

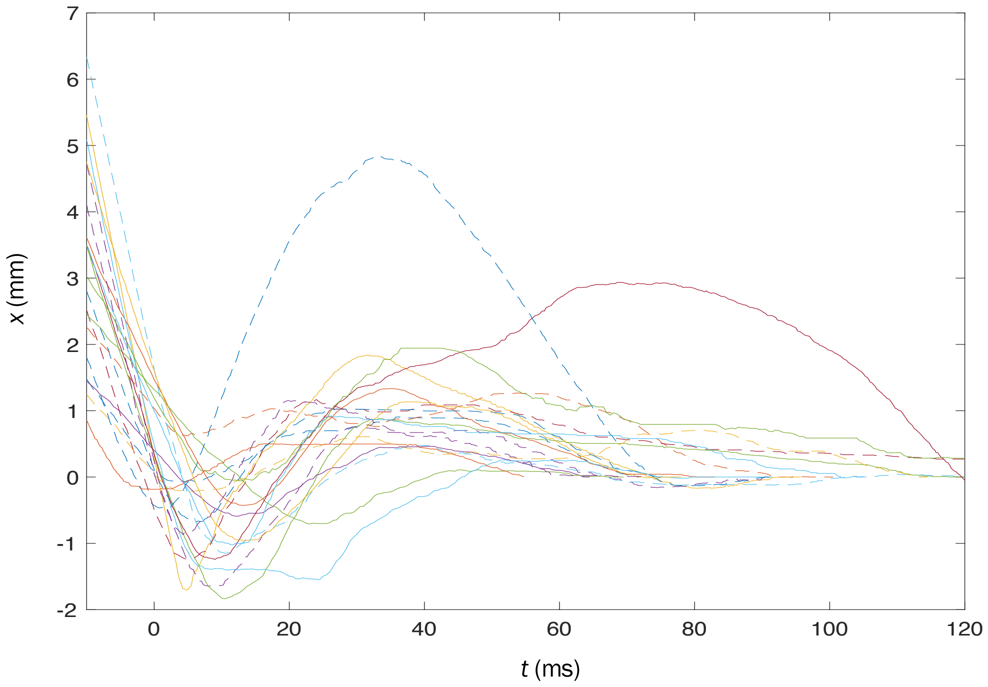

Figure S1: Normal-to-substrate displacement for all 20 analyzed landings without smoothing. The tracked point on the mosquito is the interface of the proboscis with the head. The final resting position the tracked point corresponds to $x = 0$. First contact of any portion of the mosquito with the landing surface corresponds to $t = 0$. Dashed-curves indicate the proboscis is the first member to contact the substrate, while solid lines indicate tarsi initiate contact.

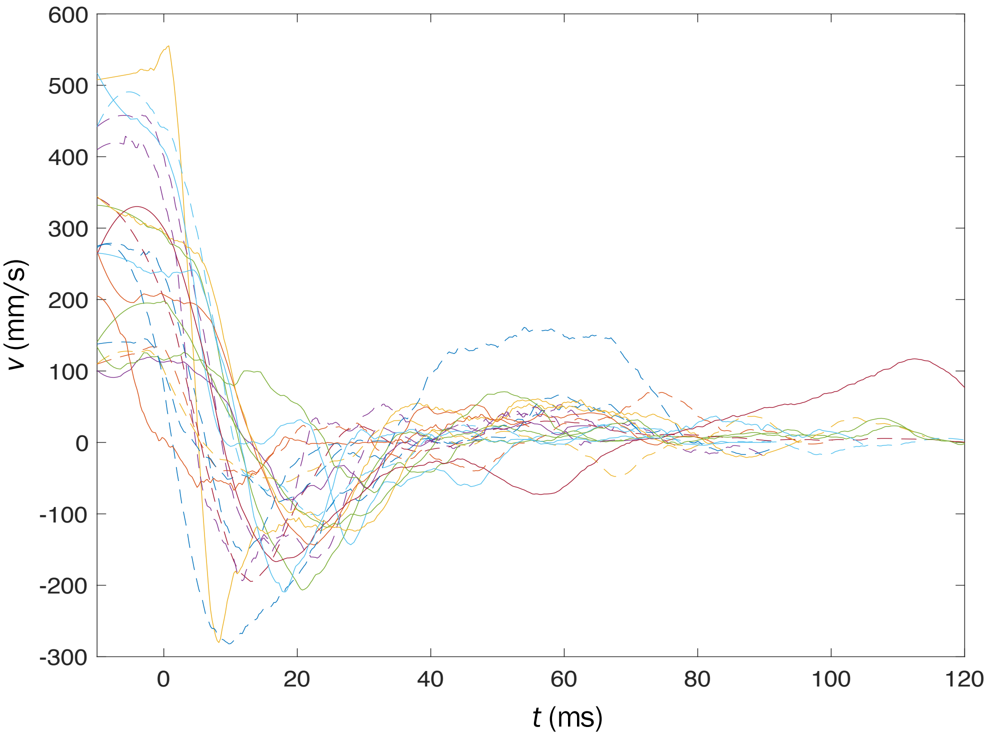

Figure S2: Normal-to-substrate velocity for all 20 analyzed landings. The tracked point on the mosquito is the interface of the proboscis with the head. First contact of any portion of the mosquito with the landing surface corresponds to $t = 0$. Dashed-curves indicate the proboscis is the first member to contact the substrate, while solid lines indicate tarsi initiate contact. Curves are smoothed with a Savitzky-Golay filter at 10% span.
